# Senile Osteoporosis: The Involvement of Differentiation and Senescence of Bone Marrow Stromal Cells

**DOI:** 10.3390/ijms21010349

**Published:** 2020-01-05

**Authors:** Abdul Qadir, Shujing Liang, Zixiang Wu, Zhihao Chen, Lifang Hu, Airong Qian

**Affiliations:** 1 Laboratory for Bone Metabolism, Key Laboratory for Space Biosciences and Biotechnology, School of Life Sciences, Northwestern Polytechnical University, Xi’an 710072, Chinaliangsj@mail.nwpu.edu.cn (S.L.); wuzx@mail.nwpu.edu.cn (Z.W.); chzhh@mail.nwpu.edu.cn (Z.C.); 2 Research Center for Special Medicine and Health Systems Engineering, School of Life Sciences, Northwestern Polytechnical University, Xi’an 710072, China; 3 NPU-UAB Joint Laboratory for Bone Metabolism, School of Life Sciences, Northwestern Polytechnical University, Xi’an 710072, China

**Keywords:** senile osteoporosis, bone marrow stromal cells, differentiation, senescence, treatment

## Abstract

Senile osteoporosis has become a worldwide bone disease with the aging of the world population. It increases the risk of bone fracture and seriously affects human health. Unlike postmenopausal osteoporosis which is linked to menopause in women, senile osteoporosis is due to aging, hence, affecting both men and women. It is commonly found in people with more than their 70s. Evidence has shown that with age increase, bone marrow stromal cells (BMSCs) differentiate into more adipocytes rather than osteoblasts and undergo senescence, which leads to decreased bone formation and contributes to senile osteoporosis. Therefore, it is necessary to uncover the molecular mechanisms underlying the functional changes of BMSCs. It will benefit not only for understanding the senile osteoporosis development, but also for finding new therapies to treat senile osteoporosis. Here, we review the recent advances of the functional alterations of BMSCs and the related mechanisms during senile osteoporosis development. Moreover, the treatment of senile osteoporosis by aiming at BMSCs is introduced.

## 1. Introduction

The word “osteoporosis” means “porous bone”, which is actually a worldwide metabolic bone disorder with high incidence. It is characterized by decreased bone mass, increased bone fragility and deteriorated microstructural bone tissues [1]. It occurs due to the imbalance between bone formation and bone resorption [2]. Although it has been observed in all races, gender and age groups, but more commonly found in women and older people [3]. It remains hidden until being revealed as a disorder through bone fractures, due to minor strokes [4]. It is considered amongst the most common human diseases associated with bone fractures and other severe secondary major health problems. According to the recent report of National Osteoporosis Foundation (NOF), every second woman and fourth man worldwide over the age of 50 years will encounter bone fracture, due to osteoporosis in their remaining lives.

Osteoporosis is generally divided into two forms, primary osteoporosis and secondary osteoporosis. The primary osteoporosis mainly contains three categories, juvenile, postmenopausal, and senile osteoporosis, while the secondary osteoporosis is mainly caused by a large number of diseases and medications [5]. The primary osteoporosis is more common than the second one, and the senile osteoporosis has become one significant health concern of the world as it is an age-related disorder that occurs in people’s 70s, leading to the attenuation of both the cortical and trabecular bones [6].

**Figure 1:**
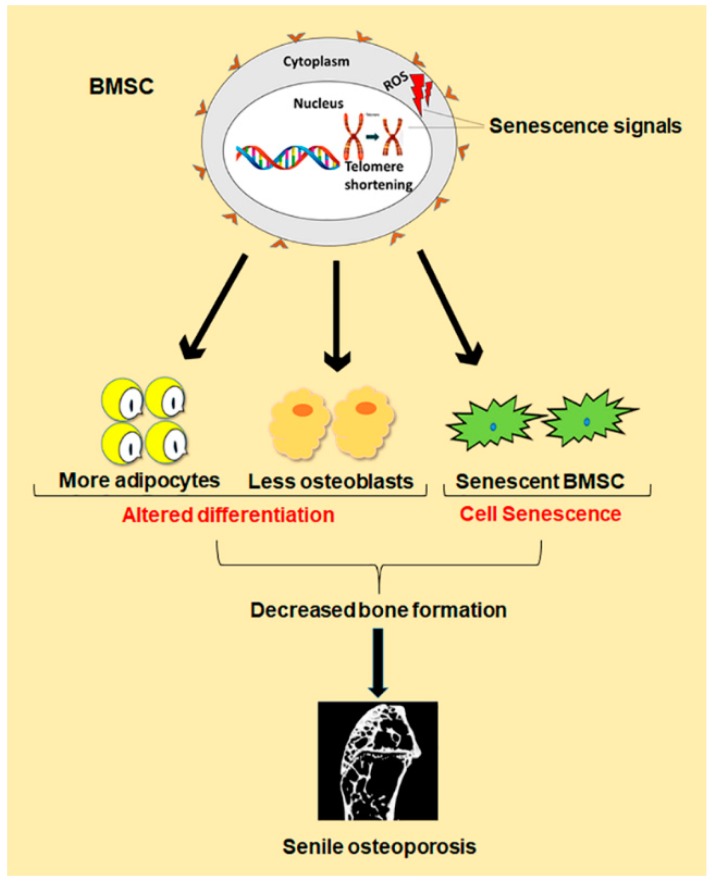
Schematic diagram of differentiation and senescence of bone marrow stromal cells (BMSCs) during senile osteoporosis.

Besides the imbalance between bone formation conducted by osteoblasts and bone resorption conducted by osteoclasts, evidence demonstrates that changes in number and function of bone marrow stromal cells (BMSCs) are also one key cause for senile osteoporosis [7]. Study shows that BMSCs normally differentiate in a proper manner into osteoblast, chondrocytes and adipocytes, but during old ages, there is comparatively less differentiation of BMSCs into osteoblast than adipocytes. Such a shift in cell differentiation of BMSCs results in reduced bone formation, which contributes to senile osteoporosis (Figure 1) [8]. The underlying mechanism behind this abnormal decision in old ages is still under investigation. However, some achievements have been made in the form of identification of peroxisome proliferator-activated receptor γ (PPARγ) and core binding factor α1 (CEBPα/β/δ) as master regulators of differentiation toward adipogenesis, while Osterix and runt-related transcription factor 2 (Runx2) toward osteogenesis [9]. In addition, recent evidence demonstrates that the senescence of BMSCs is also one important cause of senile osteoporosis (Figure 1). Cellular senescence was first discovered by Hayflick in the 1960s, which is a phenomenon where the cells halt to divide in response to various stresses causing DNA damage, and begin to secrete chemokines, cytokines, and extracellular matrix proteins, creating a toxic microenvironment called senescence-associated secretory phenotype (SASP) [10]. Such toxicity of SASP affects neighboring normal cells, resulting in further senescent cells accumulation, and thus, damage the residing tissue [11]. The expression of senescence biomarker p16Ink4a is also enhanced [12]. Cellular senescence has been demonstrated to play a crucial role in age-related pathologies, such as atherosclerosis, type II diabetes, Alzheimer’s, and Parkinson’s diseases [13]. Like the senescence of other cells associated with age-related pathologies, the exact mechanism behind BMSCs senescence during senile osteoporosis is still unclear. However, telomere shortening, oxidative stress and some genetic and epigenetic regulations have been found to contribute to BMSCs senescence during senile osteoporosis [14]. Therefore, both abnormal differentiation and senescence of BMSCs lead to the reduced number of osteoblasts in old ages, which result in decreased bone formation, thus, cause senile osteoporosis.

To date, numerous medicines have been used to treat senile osteoporosis, but there are still some limitations, due to their side effects [15,16,17,18,19]. Therefore, in order to find out proper treatments, it is the focus of new era cell-based therapy research to uncover the molecular mechanisms behind the differentiation and senescence of BMSCs. Here, we summarize the recent advances of the functional alterations of BMSCs during senile osteoporosis along with regulatory mechanisms behind their differentiation and senescence. Moreover, we also discuss the various therapies that can be used to treat senile osteoporosis by aiming at BMSCs. It will help the researchers to boost their knowledge in understanding the development of senile osteoporosis and provide them with guidance for future research on osteoporosis treatment.

## 2. Bone Marrow Stromal Cells (BMSCs) and Function Alterations of BMSCs in Senile Osteoporosis

BMSCs are MSCs located in the bone marrow and have multiple differentiation potentials to become osteoblasts, adipocytes, or chondrocytes, which play an important role in maintaining normal bone stability. It has been demonstrated that the altered proliferation and differentiation of BMSCs is one main cause of senile osteoporosis [20]. Moreover, recent evidence reveals that cellular senescence of BMSCs also contributes to senile osteoporosis [21,22]. Here, we focus on the role of altered differentiation of BMSCs and senescence of BMSCs in senile osteoporosis development.

### 2.1. Differentiation of BMSCs and Senile Osteoporosis

BMSCs differentiation is controlled by various biological, chemical and physical factors. They have the ability to differentiate into osteoblast, chondrocytes and adipocytes, which are responsible for making bones, cartilages and adipose tissues. Unfortunately, in old ages, BMSCs start to differentiate into fewer osteoblasts, but more adipocytes, which is one main cause of senile osteoporosis [23]. Justesen et al. have reported that in older people, the number of osteoblasts decreases, while that of adipocytes increases, ultimately resulting in reduced bone mineral density [24]. Similarly, Verma et al. have found that during senile osteoporosis, the rate of adipogenesis increases, while that of osteogenesis decreases [25]. Moreover, it has been demonstrated that BMSCs isolated from old mice show the high efficiency of differentiation into adipocytes as compared to osteoblasts and vice versa [26].

### 2.2. Senescence of BMSCs and Senile Osteoporosis

Like other cells, BMSCs also follow senescence, as their primary culture does not grow infinitely, but in a limited fashion. Senescence is considered to be strongly related to aging and age-related disorders like senile osteoporosis [27]. Therefore, in a study that designed a regenerative therapeutic model, Olivia et al. observed younger BMSCs more efficient than their older counterparts [28]. Moreover, during the old ages, BMSCs not only lose their functional and regenerative abilities, but also meet with replicative senescence, thus, boosting inflammation and cancer progression [29].

BMSCs are constantly affected by various exogenous and endogenous factors. These factors regulate BMSCs either to proliferate, die, or undergo a permanent cell cycle arrest [30]. Factors that lead the BMSCs and other cells to senescence include telomeres shortening, genotoxic stresses/DNA damage, strong mitogenic signals, oxidative stress, and distortions in chromatin organization [29,31,32,33]. Actually, all of these factors have the ability to elicit a DNA damage response (DDR). The DDR then stimulates p53 and its target genes p21 and p16, which ultimately initiate and maintain cellular senescence [34]. DDR also increases the level of zinc finger transcription factor GATA-binding factor 4 (GATA4), which stimulates nuclear factor kappa-light-chain-enhancer of activated B cells (NF- kB) and SASP to cause senescence [35]. SASP also induce the other neighboring cells into senescence until the whole tissue is damaged [36,37]. Therefore, senescence is also considered a persistent DNA damage response activation. In addition, with the occurrence of senescence, BMSCs are faced with the decreased potential of differentiation into osteoblasts versus adipocytes lineages [38,39]. Immunoregulatory activity is also altered, due to reduction in lymphocytes proliferation inhibition, impaired migration ability and increased proinflammatory cytokine secretion [39]. Furthermore, the increase in IL6, IL8 and galectin secretion is increased in senescent BMSCs during senile osteoporosis, which promotes tumorigenesis [40,41].

Although BMSCs are pushed into irreversible growth arrest during senescence, being metabolically active, they assume large, granular and flat shapes with increased β-galactosidase expression [42,43,44]. Despite their disorganized internal environment, they are still potentially viable and resistant to apoptosis [45].

## 3. Molecular Mechanisms Regulating Differentiation and Senescence of BMSCs during Senile Osteoporosis

During old ages, the shift of differentiation into adipocytes rather than osteoblast or become senescence of BMSCs both contribute to senile osteoporosis. Therefore, it is necessary to elaborate on the underlying molecular mechanisms. Here we primarily discuss the various intracellular biological factors like transcriptional factors, signaling pathways and epigenetic regulations. Moreover, we also introduce the various chemical and physical factors that affect BMSCs either positively or negatively, like exercise, excessive fat diet and radiations.

### 3.1. Transcription Factors

Transcription factors play an important role in regulating BMSCs to differentiate into osteoblasts or adipocytes, or undergo senescence. They are involved in regulating the expression of different genes that are responsible for initiating and promoting differentiation and senescence in BMSCs.

#### 3.1.1. Transcription Factors Involved in Osteogenic Differentiation of BMSCs

A series of transcription factors have been discovered to play major regulatory roles in osteogenic differentiation of BMSCs. Besides Runx2 and Osterix, two key osteogenic transcription factors, there are some other transcription factors that have been demonstrated as important regulators in differentiation of BMSCs into osteoblasts including β-catenin, homeobox C7 (HOXB7), core binding factor α1 (CBF-1α), forkhead box C2 (FOXC2), tumor necrosis factor-α (TNF-α), homeobox A2 (HOXA2), yes-associated protein (YAP) and bone morphogenic factor 9 (BMP9) [46].

Runx2 also known as core binding factor α1 (Cbfa1) is an important transcription factor involved in osteogenic differentiation of BMSCs. Its activation has been recognized as an initiation signal for the commitment of BMSCs to osteogenesis [47]. Actually, it induces osteogenesis both in vivo and in vitro by binding with the cis-element of the osteocalcin gene and initiates the transcription of some osteogenic genes [48]. Moreover, Yang et al. have discovered that under hypoxic condition, Runx2 is downregulated in human mesenchymal stem cells by a transcriptional repressor called twist-related protein (TWIST), which is a downstream target of hypoxia inducible factor 1-alpha (HIF-1α). They have demonstrated that during hypoxia, TWIST as a transcriptional repressor binds with the E-Box on the promoter of type 1 Runx2, which further inhibits the expression of type 2 Runx2 and other osteogenically important target genes, thus, results in impaired osteogenesis [49]. However, Jiang et al. have reported that in aging, the expression of Runx2 is immensely decreased, leading to impaired osteogenesis and bone loss [50]. Osterix, also named as Sp7, belongs to specificity protein 1 family (Sp1) and is another important transcription factor responsible for osteogenic differentiation of BMSCs. Nakashima et al. for the first time identified that Osterix functions downstream of Runx2, and there is no bone formation in mice deficient of Osterix [51]. Recently, Querques et al. identified a novel osteogenic transcriptional factor named osteoblast inducer 1 (ObI-1). They reported that knockdown of Obl-1 in BMSCs impaired osteogenesis with a poor expression of osteogenic markers, while its overexpression enhanced osteogenesis, as well as higher expression of osteogenic markers [52] (Table 1).

#### 3.1.2. Transcription Factors Involved in Adipogenic Differentiation of BMSCs

To date, a range of transcriptional factors has been identified to participate in adipogenic differentiation in BMSCs. The most well-known transcription factor is PPARγ. In addition, early B cell factor-1 (EBF-1), Twist-1, Twist-2, CCAAT/enhancer binding protein α (C/EBPα), chicken ovalbumin upstream promoter transcription factor II (COUP-II), PR domain containing 16 (PRDM16), sex determining region Y-box 2 (Sox2) and octamer-binding transcription factor 4 (Oct4) also play roles in regulating adipogenic differentiation of BMSCs [46]. Forkhead transcription factor 1 (Foxa1), GATA-binding factor 2 (GATA2) and homeobox C8 (HOXC8) transcription factors have shown an inhibitory role in regulating differentiation of MSCs into adipocytes [53,54,55].

PPARγ belongs to the nuclear receptor (NR) superfamily of ligand-activated transcription factors that regulates the genes involved in adipocyte differentiation of BMSCs [56]. It has been demonstrated that upregulation of PPARγ promotes adipogenesis in BMSCs and vice versa [57]. There are two isoforms of PPARγ, PPARγ1 and PPARγ2, both of which are prominently expressed and differentially regulated during adipogenesis [58]. Interestingly, Yu et al. reported that PPARγ1 played a lesser role in adipogenesis compared to PPARγ2, because knockdown of C/EBPα inhibited PPARγ2, but not PPARγ1 [59]. However, it has been demonstrated that in old ages, the expression of PPARγ increases, hence, promotes adipogenesis and suppresses osteogenesis [50]. Moreover, EBF-1 is another transcription factor that plays a crucial role in the adipogenic differentiation of BMSCs [60] (Table 1).

#### 3.1.3. Transcription Factors Involved in Senescence of BMSCs

During aging, BMSCs become senescent, and transcription factors are involved in this process. Nuclear factor erythroid 2-related factor 2 (NRF2) is downregulated in senescent BMSCs [50,61]. Actually, NRF2 regulates several antioxidant responsive element (ARE)-dependent genes, which express the required antioxidant, thus, ensuring the survival of BMSCs during oxidative damage [62]. Moreover, it has been reported that forkhead box protein P1 (FOXP1) level is also decreased with age in both human and murine BMSCs, which results in bone loss [63] (Table 1).

### 3.2. Signaling Pathways

To date, several signaling pathways have been reported to be involved in the regulation of BMSCs differentiation and senescence. Bone morphogenic protein (BMP), wingless-type MMTV integration site (Wnt) and Notch signaling pathways are critically important for differentiation of BMSCs, while p53/p21 and p16/Rb are important for BMSCs senescence. Apart from these, other signaling pathways like Hedgehogs (Hh), neural epidermal growth factor-like (NEL)-like protein 1 (NELL-1), insulin-like growth factor-I (IGF-I) and fibroblast growth factors (FGFs) also plays an important role in BMSCs differentiation and senescence.

#### 3.2.1. Signaling Pathways Involved in Differentiation of BMSCs

##### BMP Signaling

BMPs belong to TGF*β*1 family that is widely involved in regulating various BMSCs cellular processes like proliferation and differentiation [64]. To date, more than 20 different BMPs have been recognized to be involved in BMSCs differentiation, of which BMP2 and BMP7 have been approved by FDA for use in bone regeneration and other orthopedic applications [65] (Table 2).

The BMP signaling is normally considered as a dual role player in controlling adipogenic and osteogenic differentiation of BMSCs [66]. However, different BMP functions in different ways, and some are also dependent on the quantity of dose. BMP4 can induce adipogenesis of BMSCs [67]. High dose of BMP2 promotes osteogenesis and chondrogenesis in C3H10T1/2, while low dose induces adipogenesis in the same cells [68].

**Figure 2:**
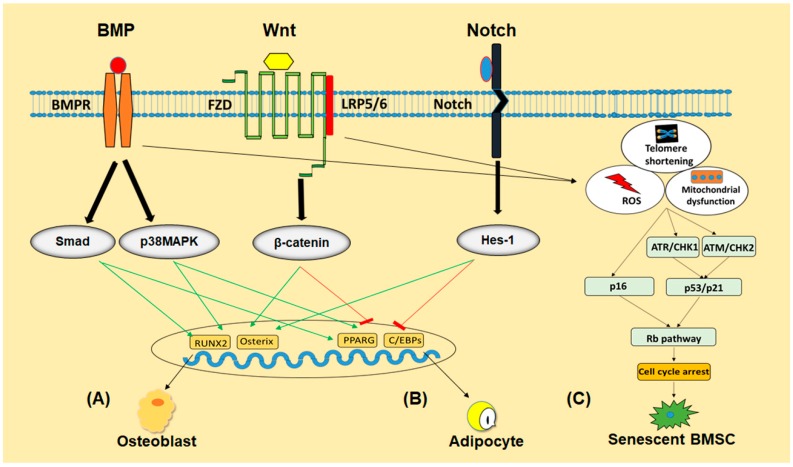
The schematic program of signaling pathways involved in regulating differentiation and senescence of BMSCs. BMP, Wnt and Notch signaling pathways regulate BMSCs differentiation into osteoblast (**A**) or adipocyte (**B**) either by promoting or inhibiting their respective transcriptional factors. Telomeres shortening, accumulation of ROS or mitochondrial damage activate p53/p21 and p16/Rb pathways in BMSCs to push them into senescence (**C**).

BMPs induce their effects by interacting with the cell surface BMP receptors (BMPRs) including BMPR-I and BMPR-II and activates canonical Smad-dependent pathways (BMP ligands, receptors, and Smads) or non-canonical Smad-independent signaling pathway (p38 mitogen-activated protein kinase (MAPK) pathway) [69,70]. Stimulation of these important pathways results in the expression of Runx2/Cbfa1 and PPAR*γ*, whose altered levels directly regulate BMSCs differentiation [71] (Figure 2).

##### Wnt Signaling

Wnt signaling pathway is an evolutionarily conserved pathway that has been recognized to perform a critical role in cell fate determination, cell proliferation and differentiation [72]. Therefore, inappropriate modifications in Wnt signaling will cause severe disorders like cancer, osteoporosis and congenital disabilities [72,73]. Wnt family consists of a range of secreted glycoproteins modified with lipids, which restrict them to function as short-range cellular signals [74]. Wnt signaling pathway can be classified as canonical Wnt signaling and non-canonical Wnt signaling, of which canonical Wnt signaling is well-established in regulating bone formation both in human and in animals. Being dependent on β-catenin, canonical signaling is also called Wnt/β-catenin signaling. Wnt ligands function by interacting with 7-transmembrane domain-spanning Frizzled receptor (FZD) and lipoprotein receptors-related protein 5/6 (LRP5/6) coreceptors, thereby stabilizing β-catenin by saving it from phosphorylation and degradation which translocate readily into the nucleus to regulate various target genes expression [75] (Figure 2).

Wnt signaling plays a crucial role in BMSCs differentiation by promoting osteogenesis and inhibiting adipogenesis. Wnt3a can induce osteogenic differentiation by activating TAZ through PP1A-mediated dephosphorylation [76]. Overexpression of Wnt10b was found to increase postnatal bone formation by promoting osteoblastogenesis [77]. Increase in adipocytes number during old ages is also considered to be related to the decline of Wnt10b [78]. Moreover, Arango et al. also found promotion in adipogenesis in myometrium with conditional deletion of β-catenin in the mesenchyme of the developing mouse uterus in vivo [79] (Table 2).

##### Notch Signaling

The notch signaling pathway is involved in regulating adipogenic and osteogenic differentiation of BMSCs, either through directly targeting the respective genes or communicating with other signaling pathways. It consists of Notch and notch ligands, which are transmembrane proteins involved in cell differentiation [80] (Figure 2). In addition to its function in adipogenic differentiation, this signaling has been found to suppress osteogenic differentiation as well [80]. On the other hand, Shimizu et al. have reported that Notch signaling can induce osteogenesis through interaction with BMP2 signaling [81].

##### Other Signaling Pathways

Other signaling pathways like Hedgehog, NELL-1, FGFs and IGF-I are also involved in the differentiation of BMSCs. Hedgehog signaling is another pathway involved in BMSCs differentiation that promotes osteogenesis and suppresses adipogenesis. Spinella-Jaegle et al. reported that Hedgehog signaling was found with inhibited adipogensis and promoted osteogenesis at the same time [82]. NELL-1 signaling also induces osteogenesis associated with antiadipogenic effects in BMSCs [83,84]. Furthermore, FGFs have been reported to play an important role in both adipogenic and osteogenic differentiation of BMSCs [85,86]. Moreover, IGF-I signaling is also known for its critical role in bone remodeling and adipogenic differentiation of BMSCs [87] (Table 2).

#### 3.2.2. Signaling Pathways Involved in Senescence of BMSCs

##### p53/p21 and p16/Rb

p53/p21 and p16/Rb (tumor suppressor retinoblastoma protein, Rb) are the two interrelated key pathways involved in regulating senescence of BMSCs. In addition, some other pathways like TGF-β, Wnt, MAPK, PI3K/AKT/mTOR, Notch and FGFs also function in controlling senescence of BMSCs.

p53/p21 and p16/Rb signaling pathways are actually regulated in responding to some phenomena causing DNA damage, such as telomeres shortening, reactive oxygen species (ROS) accumulation. Shortening of telomeres occurs after every cell division, ultimately reach to “Hayflick limit”, which deprives the cells of further division [88]. This phenomenon of telomeres shortening elicits DNA damage response which contains multiple signaling events centered on p53/p21 and p16/Rb pathways [89]. Both of these pathways are based on anti-proliferative mechanisms that halt the cells from dividing further, allowing the cells to repair themselves. However, when the DNA damage is exceeding in the cells, these phenomenon forward them to senescence [90]. Similarly, immoderate accumulation of ROS, such as superoxide anion, hydrogen peroxide and hydroxyl radical also trigger DNA damage response, followed by p16/Rb and p53 pathways, which ultimately lead the cells to senescence [91] (Figure 2).

##### Other Signaling Pathways

BMP signaling is also involved in inducing senescence in BMSCs through contributing to the production of ROS and triggering DNA damage response [92]. Wnt/β-catenin signaling pathway being actively involved in BMSCs differentiation also participates in inducing senescence through interacting with p53/p21 pathway to produce ROS [93]. Moreover, other signaling pathways including MAPK, PI3K/AKT/mTOR, Notch, FGFs and Hipo have been reported to play a crucial role in causing senescence in BMSCs as well [94] (Table 2).

### 3.3. Epigenetic Regulations

Epigenetic regulations play important roles in the differentiation and senescence of BMSCs. During transcription, transcriptional factors are not only involved in regulating gene expressions, but also in correlation with epigenetic factors. Mutations in such epigenetic factors even result in severe disorders like osteoporosis and cancer. Therefore, it is important to elaborate the role of epigenetic regulations including DNA methylation, histone modifications, and epigenetic regulators such as non-coding RNAs (e.g., MicroRNAs) in the differentiation and senescence of BMSCs.

#### 3.3.1. Epigenetic Factors Involved in Osteogenic Differentiation of BMSCs

Epigenetic regulations actually alter the binding ability of osteogenic transcriptional factors with their target promoters by changing chromatin structures. It is a recognized fact that Runx2 works as a master regulator in inducing osteogenesis in BMSCs [95]. However, its transcriptional activity is controlled by various epigenetic factors including coactivators and corepressors [96]. Osteocalcin (OC) promoter is the most widely studied promoter providing the binding sites for numerous osteogenically important transcriptional factors like Runx2 [97]. It has been reported that hypo DNA methylation and histones, H3 and H4 acetylation enhance the binding ability of *OC* promoter to osteo-inductive transcription factors [98]. Villar-Garea et al. found that very eminent hypermethylation was observed at the OC gene promoter, which was confirmed to be related to condensed chromatin structure [99]. Villagra et al. have shown the decrease in DNA methylation of OC promoter region during in vitro osteoblast differentiation of BMSCs [100]. Upregulation of bone related genes, due to mechanical loading has also been found with decreased CpG methylation [101]. Further, Shen et al. found an increased level of acetylation at H3 and H4 histones near the promoter region of OC gene during osteoblastic differentiation of BMSCs, hence, reported an absolute association between core histone and OC gene expression [98]. Apart from these, nicotinamide phosphoribosyltransferase (Nampt), absent, small, or homeotic disc1 like (Ash1l) and CCAAT/enhancer-binding protein beta (CEBPB) have also been reported to play important roles in augmenting osteogenic differentiation of BMSCs [102,103,104]. MicroRNAs being epigenetic regulators also play their roles during osteogenic differentiation of BMSCs. To date, most of the miRNAs have shown negatives effects in regulating the osteogenic differentiation of BMSCs. MicroRNAs including miR-31, miR-138, miR-204, and mir-637 have been investigated to inhibit osteogenic differentiation of BMSCs [105,106,107,108]. However, recently, Yan et al. reported that let-7c-5p, miR-181c-3p, miR-3092-3p and miR-5132-3p promoted osteogenic differentiation of mouse BMSCs [109].

#### 3.3.2. Epigenetic Factors Involved in Adipogenic Differentiation of BMSCs

Epigenetic regulations also play an important role in adipocyte differentiation. Just as osteogenic differentiation, adipogenic differentiation is a well-organized phenomenon containing transcription factors performing various functions. PPAR-*γ* is the master regulator of adipogenic differentiation of BMSCs [110]. Its activity is regulated by various epigenetic regulation. Noer et al. found certain adipogenic promoters including PPARγ, leptin, fatty acid-binding protein 4 (fabp4), and lipoprotein lipase (lpl) hypomethylated by investigating isolated adipose stromal cells, hence, showed the importance of epigenetic activity, such as methylation in adipogenesis [111]. Bowers et al. treated C3H/10T1/2 cells with 5-azacytidine that induce them into adipocytes spontaneously, due to the proper demethylation and expression of BMP4 gene [112]. Similar to DNA methylation, histone methylation is also very necessary in adipogenic differentiation of BMSCs, of which H3 lysine 4 (H3K4) is of prime importance. 3T3-L1 fibroblast cells treated with low-dose of methyltransferase inhibitor methylthioadenosine showed a quite significant decline in adipocyte differentiation, which is due to the removal of epigenetic sign from the promoters, thus, proved the important role of histone modification in regulating adipogenesis [113]. H3K4me2, which is considered to be the active mark of transcription has been found on the promoter region of certain important adipogenic genes that are *adiponectin*, *glut4*, and *lep* during commitment [113]. Moreover, the decreased level of HDACs has been identified to be associated with adipogenesis and vice versa. Unphosphorylated retinoblastoma (Rb) protein has been found to repress adipogenesis by recruiting HDAC3 to the promoters of *PPARγ* gene [114].

Apart from these, microRNAs also function in regulating adipogenic differentiation of BMSCs. Qadir et al. have identified that miR-124 promotes adipogenesis of BMSCs by suppressing the expression of a pro-osteogenic transcription factor Dlx5 [115]. Similarly, miR-30, miR-204, miR-211, miR-320 have been recognized to induce adipogenesis of BMSCs by targeting Runx2 [116,117]. Furthermore, miR-188 has been found to play a role in fat accumulation and bone loss, especially during aging [118].

#### 3.3.3. Epigenetic Factors Involved in Senescence of BMSCs

In addition to the other factors, epigenetic changes are also involved in causing senescence of BMSCs. It has been identified that the DNA methylation levels slowly decrease with time in cell culture [119]. So et al. have reported that DNA methyltransferase (DNMT) level decreases during replicative senescence of BMSCs, thus, leads to hypomethylation, which is a well-known characteristic of senescent cells. Furthermore, they demonstrated that DNMTs played a role in inducing senescence not only through DNA methylation status, but also by activating or inactivating histone marks in genomic regions of Polycomb group (PcG)-targeting miRNAs and p16^INK4A^ and p21^CIP1/WAF1^ promoter regions [120]. Histone modifications, such as acetylation and methylation, also contribute to senescence in BMSCs. Histone deacetylases (HDACs) are mostly downregulated during senescence of BMSCs, which result in the low expression of the polycomb group genes and high expression of Jumonji domain containing three proteins, thus, controlling cell cycle [121]. Moreover, Fernandez et al. observed the active chromatin mark H3K4me1 enriched with hypomethylated regions during cellular senescence of BMSCs, hence, showing the importance of histone methylation in senescence [122]. MicroRNAs also participate in the induction and maintenance of senescence through regulating various parts of the cell cycle or nuclear organization. To date, about 45 miRNAs have been identified to be involved in replicative senescence of BMSCs [123]. The let-7 family of miRNAs is of prime importance in inducing senescence through transcriptional gene silencing of E2F-regulated proliferation promoting genes [124]. Lal et al. have reported that miR-24 promotes p16-dependent senescence pathway by targeting and downregulating p16 mRNA, which leads to replicative senescence of BMSCs [125]. Wagner et al. have found the upregulation of miR-369-5p, miR-29c, and let-7f during replicative senescence [126]. In addition, miR-34a has been identified to downregulate the expression of class-III histone deacetylase silent information regulator 1 (SIRT1), thus, resulting in the increased level of p21 levels, triggering senescence [127]. MiR-195 and miR-495 have also played roles in causing senescence in BMSCs [128,129].

### 3.4. Other Factors

Besides transcription factors, signaling pathways and epigenetic regulations, there are some other external or internal chemical, physical and biological factors that induce differentiation and senescence of BMSCs.

#### 3.4.1. Chemical Factors

Normally, for osteogenic differentiation, BMSCs are cultured in a medium containing L-ascorbic acid (AA), β-glycerophosphate (βGP) and Dexamethasone. Combination of these chemicals has the ability to initiate collagenous extracellular matrix formation, which upregulates the expression of osteogenic indicators alkaline phosphatase (ALP) and OC [130]. Similarly, the combination of chemicals, such as isobutylmethylxanthine (IBMX), insulin and dexamethasone results in adipogenic differentiation of BMSCs in culture [131]. It has been reported that IBMX and dexamethasone initiate adipogenic differentiation, while the insulin is associated with the uptake of glucose to synthesize triglycerides in adipocytes [132,133]. Moreover, a low dose of pesticides mixtures and high glucose levels have been reported to induce senescence by triggering the formation of ROS and upregulating autophagy in BMSCs, respectively [134,135].

#### 3.4.2. Physical Factors

Some physical factors, such as radiations and mechanical stimuli also contribute importantly to the differentiation and senescence of BMSCs. It was observed that a range radiations (0–16 Gy) applied on human pluripotent cells and BMSCs promoted adipogenesis and declined osteogenesis [136]. Furthermore, Alessio et al. have found that treatment of BMSCs with a low dose of radiations induces cell senescence [137]. Being mechanosensitive, BMSCs differentiation is also regulated by certain mechanical factors, such as exercise, vibration, and microgravity. It has been reported in several studies that exercise enhances osteogenesis and reduces adipogenesis in BMSCs. Interestingly, Menuki et al. have shown that 28 days of climbing exercise increased bone volume and osteoblast number and decreased bone marrow adipocyte number in a 8-week-old male mice [138]. Vibration at low frequency has been identified with a positive effect on bone formation and negative effect on adipogenesis in BMSCs [139,140]. Moreover, Zayzafoon et al. found a decrease in osteoblastic differentiation and increase in adipogenic differentiation of human BMSCs in response to modelled microgravity, hence, showed the negative effect of microgravity on bone formation [140]. However, there is still no data available regarding mechanical stimuli to induce senescence in BMSCs.

#### 3.4.3. Biological Factors

Other biological factors like aging and metabolism, also regulate BMSCs differentiation and senescence. During aging, BMSCs differentiate more into adipocytes and less into osteoblasts, resulting in bone loss. Moerman et al. found increased expression of PPARγ in aged BMSCs, which is considered as the master regulator of adipogenesis [26]. Altered mitochondrial metabolism and generation of ROS are also main factors regulate the adipogenic differentiation of BMSCs [141]. High uptake of glucose and fat diet are also responsible for promoting adipogenic differentiation and decreasing osteogenic differentiation of BMSCs [142]. Moreover, oxidative stress has been reported to induce senescence both in vitro and in vivo in BMSCs [143].

## 4. Treatment of Senile Osteoporosis by Aiming at BMSCs

In old ages, BMSCs either differentiate into more adipocytes than osteoblasts or assume senescence, which ultimately results in senile osteoporosis. Therefore, in order to treat senile osteoporosis, it is required to use the strategies in what BMSCs can be stimulated either to differentiate into more osteoblasts than adipocytes or be eliminated their senescence. To date, numerous molecules including parathyroid hormone (PTH 1–84) or only its N-terminal fragment teriparatide (PTH 1–34), bisphosphonates, tetracycline, cationic peptides and antibodies like denosumab and romosozumab have been used in the treatment of senile osteoporosis [15,16,17,18,19]. However, most of them are limited either, due to their severe side effects or inhibition of just bone resorption without decreasing bone regeneration. Therefore, in order to reduce such limitations, there is the need of using cell-based therapy strategy, for which BMSCs can act as an ideal cell source, due to their self-renewing and differentiation ability into various types of cells. In addition, easy isolation with high yields from different tissues, and immunosuppressive and immunoprivileged properties of BMSCs also make them the preferable cell source in cell-based therapies [144].

In order to treat senile osteoporosis, several researchers have reported the successful transplantation of BMSCs using animal models. Transplanted BMSCs, serve in bone formation either by allocating damaged areas to differentiate into osteoblasts or assume paracrine mode, due to which they secrete specific growth factors to make a favorable environment for the nearby cells to repair the degenerative tissue [145]. Ichioka et al. injected normal allogeneic BMSCs intra bone marrow into the senescence accelerated mouse prone 6 (SAMP6) mice, naturally prone to senile osteoporosis in their early lives. They demonstrated that the injected normal BMSCs were able to prevent the senile osteoporosis in SAMP6 mice with an increase in trabecular bone mass and decline in BMD loss [146]. Takada et al. also treated osteoporosis after it occurred in aged SAMP6 mice by injecting normal allogeneic BMSCs locally into their bone marrow. After the clinical examinations, no signs of senile osteoporosis were found, hence, succeeded in proving their hypothesis [147]. In another experimental procedure, when BMSCs isolated from healthy rats were injected into the bone marrow of femurs of osteoporotic female ovariectomized rats, a quite increase in the bone mass of femur was observed after examination [148]. Similarly, Kiernan et al. also found an increase in bone formation when they injected systemically normal allogeneic BMSCs into the bone marrow of senile osteoporotic mouse model, giving a clue towards their applications against human senile osteoporosis [149].

Certain factors, microRNAs and long non-coding RNAs have also been recognized to play significant roles in treating senile osteoporosis by stimulating BMSCs to differentiate into more osteoblasts than adipocytes. Suppression of ectopic viral integration site‑1 (Evi1) gene through RNA interference in rat BMSCs resulted in increased osteogenesis and decreased adipogenesis, suggesting Evi1 as a potent target for targeting osteoporosis [150]. Huan et al. have reported enhancer of zeste homology 2 (EZH2) factor as a competent therapeutic target for enhancing bone formation during osteoporosis as its suppression led to increased osteogenesis rather than adipogenesis [151]. Recently, Zhou et al. uncovered the role of orcinol glucoside (OG), a constituent of traditional Chinese medicine, in promoting bone formation. They reported that OG was able to revert the BMSCs differentiation fashion of more into adipocytes than osteoblasts in old ages through Wnt/β-catenin signaling pathway, thus, may act as a novel therapeutic agent against senile osteoporosis [152]. Chang-Jun et al. found the increased bone formation and decreased fat accumulation after injecting aptamer-antagomiR-188 into the bone marrow of osteoporotic aged mice. The aptamer-antagomiR-188 actually inhibited miR-188, whose overexpression is actually responsible for reducing osteogenesis and increasing adipogenesis [118]. Let-7, a miRNA family, has also been distinguished to promote osteogenesis and decline adipogenesis in BMSCs [153]. Very recently, Zhao et al. demonstrated that miR-21 possesses the ability to stimulate the osteogenic differentiation of BMSCs by finding the role of miR-21 inhibitor in inhibiting BMSCs differentiation into osteoblasts [154]. Recently, long noncoding RNA Bmncr was found as key regulator in promoting osteogenesis and inhibiting adipogenesis in mice during aging, suggesting it to be a therapeutic target against senile osteoporosis in future [155]. Chen et al. reported that overexpression of lncRNA XIST led to the inhibition of osteogenic differentiation of BMSCs in 3-week-old Sprague Dawley rats [156], thus, its inhibition through specific inhibitor can revert the phenomenon and can treat the senile osteoporosis. Most recently, Zhu et al. have identified lncRNA HOXA-AS2 as a key positive regulator in causing osteogenesis in BMSCs through NF-*κ*B signaling inactivation [157], which may act as a new therapeutic target against senile osteoporosis.

Different approaches have also been used to eliminate the senescence of BMSCs, and thus, treat senile osteoporosis. Elimination of senescent cells is of much importance regarding bone mass and strength. In order to uncover such importance, Joshua et al. used some genetic and pharmacological procedures to eliminate the senescent cells. They found that activating INK-ATTAC caspase 8 in senescent cells or treating senescent cells with JAK inhibitor or senolytics increased bone mass and bone strength in mice with the bone loss [21]. A senolytic drug, ABT263 can also reduce senescence associated factors, hence, can act as a good therapeutic drug against senile osteoporosis [158]. Gao et al. delivered tetramethylpyrazine (TMP) locally into the bone marrow of aging mice with established senescent BMSCs microenvironment, a significant reduction was found in senescent phenotype via modulating Ezh2-H3k27me3, suggesting TMP as a potent local eliminator of senescent BMSCs in age-related bone loss [159]. Sun et al. suppressed the expression of NADPH oxidase, which is mainly involved in ROS formation in BMSCs; they found a significant increase in osteoblasts differentiation of BMSCs. Moreover, they also found an increase in bone formation after treating SAMP6 mice with apocynin for three months, hence, declared as a competent therapeutic agent against age-related bone loss [160]. More recently, Zhou et al. demonstrated that resveratrol was able to attenuate senescence and promote osteogenic differentiation of BMSCs by inhibiting AMPK activation/ROS inhibition signaling pathway in aged mouse, suggesting resveratrol as a novel therapy against senile osteoporosis, due to its inhibiting effects on ROS formation in BMSCs [161].

## 5. Conclusion and Perspectives

Senile osteoporosis is an age dependent bone disorder occurring both in men and women, which has become a worldwide health concern. The functional change of BMSCs has been demonstrated to contribute to senile osteoporosis, showing as BMSCs differentiate into fewer osteoblasts, but more adipocytes and BMSCs become senescent. Besides the critical involvement of BMSCs in senile osteoporosis, BMSCs are also favorite cell source for cell therapy and have been applied for osteoporosis treatment. Therefore, uncovering the underlying mechanisms of function changes of BMSCs during senile osteoporosis is important not only for better understanding the involvement of BMSCs in senile osteoporosis, but also for manipulating them for clinical applications.

Recent findings demonstrate that numerous transcriptional factors, signaling pathways, epigenetic regulations and other factors play key roles in regulating the differentiation and senescence of BMSCs, the alteration of which contributes to senile osteoporosis. Runx2 and PPARγ are two key transcription factors that are responsible for osteogenic differentiation and adipogenic differentiation of BMSCs, respectively. Decreased Runx2 expression and increased PPARγ results in senile osteoporosis. NRF2 and FOXP1 are two transcription factors related to the senescence of BMSCs by regulating antioxidant responsive genes. They are decreased with age, thus, leads to BMSCs senescence and bone loss. BMP signaling, Wnt signaling and Notch signaling pathways all show dual roles in regulating osteogenic and adipogenic differentiation of BMSCs. They function either by targeting the downstream transcription factors, such as Runx2, PPARγ, or by cross-talking with each other. Recently, p53/p21 and p16/Rb signaling pathways have been demonstrated to be involved in the senescence of BMSCs, which is one main cause of senile osteoporosis. These signaling pathways are activated by DNA damage or ROS accumulation and finally lead to cell senescence. Besides, BMP signaling and Wnt signaling also participate in inducing senescence of BMSCs by inducing ROS, triggering DNA damage or interacting with p53/p21 signaling. Moreover, epigenetic regulation also plays important role in regulating differentiation and senescence of BMSCs. The epigenetic regulation, such as DNA methylation and histones acetylation, regulates the differentiation and senescence of BMSCs by regulating the expression of transcription factors or disturbing the binding of transcription factors to specific gene’s promoter. These findings provide an understanding of the molecular mechanisms underlying the altered differentiation and senescence of BMSCs during senile osteoporosis and provide potential targets or methods for treating senile osteoporosis.

Direct transplantation of normal BMSCs and elimination of senescent BMSCs both efficiently treat senile osteoporosis. Transplantation of normal allogeneic BMSCs into aged mice shows both prevention and treatment effects on senile osteoporosis. In addition, modification of the differentiation ability of BMSCs through targeting some genes can be applied for treating senile osteoporosis. More recently, elimination of senescent BMSCs has been demonstrated to be an effective therapeutic method for treating senile osteoporosis. All these findings strongly demonstrate that BMSCs can be applied for clinical treatment of senile osteoporosis by directly transplanting normal BMSCs, modifying differentiation of BMSCs, or eliminating senescent BMSCs. However, present findings are obtained from animal studies. Further clinical trials are needed.

In summary, the altered differentiation and senescence of BMSCs contribute to senile osteoporosis. Transplantation of normal BMSCs, modification of altered differentiation ability of BMSCs, and elimination of senescent BMSCs can effectively treat the senile osteoporosis. However, as differentiation and senescence, the two key physiological processes of BMSCs, are sometimes closely linked, there are still some questions to be investigated. How do BMSCs make a choice, to differentiate to specific cell type or undergo senescence? What regulatory factors play a primary role in regulating these processes? Moreover, for the treatment of senile osteoporosis, as present findings are from animal studies, clinical trials are needed in future. By answering these questions and conducting clinical trials, we will get a better understanding of the role and mechanisms of BMSCs in senile osteoporosis and may provide new insights for manipulating BMSCs for clinical applications.

## Figures and Tables

**Table 1:** Transcriptional factors involved in differentiation and senescence of BMSCs.

Transcriptional Factors	Function	References
Runx2	Promotes osteogenic differentiation, and inhibits adipogenic differentiation and senescence	[48,49,50]
Osterix	Promotes osteogenic differentiation	[51]
Obl-1	Promotes osteogenic differentiation	[52]
PPARγ	Promotes adipogenic differentiation and senescence, and inhibits osteogenic differentiation	[50,56,57,58,59]
EBF-1	Promotes adipogenic differentiation	[60]
NRF2	Inhibits senescence	[61,62]
FOXP	Inhibits senescence	[63]

Runx2, runt-related transcription factor 2; Obl-1, osteoblast inducer 1; PPARγ, peroxisome proliferator-activated receptor-gamma; EBF-1, early B cell factor; NRF2, nuclear factor Erythroid 2-related factor 2; FOXP, forkhead transcription factor P.

**Table 2:** Signaling pathways involved in differentiation and senescence of BMSCs.

Signaling Pathways	Functions	References
TGF-β/BMP	Controls both osteogenesis and adipogenesis in a proper manner, and also induces senescence	[66,92]
Wnt	Induces osteogenesis and inhibits adipogenesis	[77,78]
Notch	Promotes osteogenesis and inhibits adipogenesis	[80]
Hedgehog	Promotes osteogenesis and suppresses adipogenesis	[82]
NELL-1	Induces osteogenesis with antiadipogenic effects	[83]
FGFs	Control both osteogenesis and adipogenesis with equal effects	[85,86]
IGF-I	Promotes adipogenic differentiation	[87]
p53/p21	Induces senescence	[88,89,90,91]
p16/Rb	Induces senescence	[88,89,90,91]

BMP, bone morphogenic protein; Wnt, wingless-type MMTV integration site; NELL-1, Neural epidermal growth factor-like (NEL)-like protein 1; FGFs, fibroblast growth factor; IGF-I, insulin-like growth factor-I.

## Abbreviations

AA	ascorbic acid
Ash1l	absent, small, or homeotic disc1 like
Ash1l	alkaline phosphatase
AMPK	adenosine monophosphate-activated protein kinase
βGP	β-glycerophosphate
BMD	bone mineral density
BMP	bone morphogenic protein
BMSCs	bone marrow stromal cells
CEBPB	CCAAT/enhancer-binding protein beta
C/EBPα	core binding factor α1
DLX5	distal-less Homeobox 5
DDR	DNA damage response
DNMT	DNA methyltransferase
EBF-1	early B cell factor
EZH2	enhancer of zeste homology 2
Ezh2-H3k27me3	enhancer of zeste homolog2-tri-methylation of histone H3 at Lys 27
FGFs	fibroblast growth factors Foxa1
FOX	forkhead transcription factor
FZD	7-transmembrane domain-spanning Frizzled receptor
GATA2	GATA-binding factor 2
HDACs	histone deacetylases
HOXA-AS2	HOXA Cluster Antisense RNA 2
HIF1	Hypoxia-Inducible Factor 1
IBMX	isobutylmethylxanthine
IGF-I	insulin-like growth factor-I
JAK	janus kinase
LRP5/6	lipoprotein receptors-related protein 5/6
MAPK	mitogen-activated protein kinase
miRNA	microRNA
Nampt	nicotinamide phosphoribosyltransferase
NF-κB	nuclear factor kappa-light-chain-enhancer of activated B cells
NELL-1	neural epidermal growth factor-like (NEL)-like protein 1
NRF2	nuclear factor Erythroid 2-related factor 2
ObI-1	osteoblast inducer 1
OC	osteocalcin
OG	orcinol glucoside
PcG	Polycomb group
PPARγ	peroxisome proliferator-activated receptor-gamma
Rb	retinoblastoma
ROS	reactive oxygen species
Runx2	runt-related transcription factor 2
SAMP6	senescence accelerated mouse prone 6
SASP	senescence-associated secretory phenotype
SIRT1	silent information regulator 1
Sox2	sex determining region Y-box 2
TGF-β	transforming growth factor-β TGF-β
TMP	tetramethylpyrazine
TNF-α	tumor necrosis factor-α
Twist	twist-related protein
Wnt	wingless-type MMTV integration site
Xist	X-inactive specific transcript
YAP	yes-associated protein 1

## References

[1] Ge D.W., Wang W.W., Chen H.T., Yang L., Cao X.J. (2017). Functions of microRNAs in osteoporosis. Eur. Rev. Med. Pharm. Sci..

[2] Florencio-Silva R., Sasso G.R., Sasso-Cerri E., Simoes M.J., Cerri P.S. (2015). Biology of Bone Tissue: Structure, Function, and Factors That Influence Bone Cells. BioMed Res. Int..

[3] Vondracek S.F., Linnebur S.A. (2009). Diagn116osis and management of osteoporosis in the older senior. Clin. Interv. Aging.

[4] Cosman F., de Beur S.J., LeBoff M.S., Lewiecki E.M., Tanner B., Randall S., Lindsay R., National Osteoporosis F. (2014). Clinician’s Guide to Prevention and Treatment of Osteoporosis. Osteoporos Int..

[5] Briot K., Roux C., Thomas T., Blain H., Buchon D., Chapurlat R., Debiais F., Feron J.M., Gauvain J.B., Guggenbuhl P. (2018). 2018 update of French recommendations on the management of postmenopausal osteoporosis. Jt. Bone Spine.

[6] Nuti R., Brandi M.L., Checchia G., Di Munno O., Dominguez L., Falaschi P., Fiore C.E., Iolascon G., Maggi S., Michieli R. (2019). Guidelines for the management of osteoporosis and fragility fractures. Intern. Emerg. Med..

[7] Kiernan J., Davies J.E., Stanford W.L. (2017). Concise Review: Musculoskeletal Stem Cells to Treat Age-Related Osteoporosis. Stem Cells Transl. Med..

[8] Infante A., Rodriguez C.I. (2018). Osteogenesis and aging: Lessons from mesenchymal stem cells. Stem Cell Res. Ther..

[9] Tang Q.Q., Lane M.D. (2012). Adipogenesis: From stem cell to adipocyte. Annu. Rev. Biochem..

[10] Nelson G., Wordsworth J., Wang C., Jurk D., Lawless C., Martin-Ruiz C., von Zglinicki T. (2012). A senescent cell bystander effect: Senescence-induced senescence. Aging Cell.

[11] Acosta J.C., Banito A., Wuestefeld T., Georgilis A., Janich P., Morton J.P., Athineos D., Kang T.-W., Lasitschka F., Andrulis M. (2013). A complex secretory program orchestrated by the inflammasome controls paracrine senescence. Nat. Cell Biol..

[12] Tchkonia T., Zhu Y., Van Deursen J., Campisi J., Kirkland J.L. (2013). Cellular senescence and the senescent secretory phenotype: Therapeutic opportunities. J. Clin. Investig..

[13] Khosla S., Farr J.N., Kirkland J.L. (2018). Inhibiting cellular senescence: A new therapeutic paradigm for age-related osteoporosis. J. Clin. Endocrinol. Metab..

[14] Li Y., Wu Q., Wang Y., Li L., Bu H., Bao J. (2017). Senescence of mesenchymal stem cells (Review). Int. J. Mol. Med..

[15] Cipriano C.A., Issack P.S., Shindle L., Werner C.M., Helfet D.L., Lane J.M. (2009). Recent advances toward the clinical application of PTH (1-34) in fracture healing. HSS J..

[16] Hilgenbrink A.R., Low P.S. (2005). Folate receptor-mediated drug targeting: From therapeutics to diagnostics. J. Pharm. Sci..

[17] Lewiecki E.M., Dinavahi R.V., Lazaretti-Castro M., Ebeling P.R., Adachi J.D., Miyauchi A., Gielen E., Milmont C.E., Libanati C., Grauer A. (2019). One Year of Romosozumab Followed by Two Years of Denosumab Maintains Fracture Risk Reductions: Results of the FRAME Extension Study. J. Bone Miner. Res..

[18] Mingozzi F., High K.A. (2011). Therapeutic in vivo gene transfer for genetic disease using AAV: Progress and challenges. Nat. Rev. Genet..

[19] Rehman Z., Zuhorn I.S., Hoekstra D. (2013). How cationic lipids transfer nucleic acids into cells and across cellular membranes: Recent advances. J. Control. Release.

[20] Hu L., Yin C., Zhao F., Ali A., Ma J., Qian A. (2018). Mesenchymal Stem Cells: Cell Fate Decision to Osteoblast or Adipocyte and Application in Osteoporosis Treatment. Int. J. Mol. Sci..

[21] Farr J.N., Xu M., Weivoda M.M., Monroe D.G., Fraser D.G., Onken J.L., Negley B.A., Sfeir J.G., Ogrodnik M.B., Hachfeld C.M. (2017). Targeting cellular senescence prevents age-related bone loss in mice. Nat. Med..

[22] Shen J., Tsai Y.-T., DiMarco N.M., Long M.A., Sun X., Tang L. (2011). Transplantation of mesenchymal stem cells from young donors delays aging in mice. Sci. Rep..

[23] Wang C., Meng H., Wang X., Zhao C., Peng J., Wang Y. (2016). Differentiation of Bone Marrow Mesenchymal Stem Cells in Osteoblasts and Adipocytes and its Role in Treatment of Osteoporosis. Med. Sci. Monit..

[24] Justesen J., Stenderup K., Ebbesen E., Mosekilde L., Steiniche T., Kassem M. (2001). Adipocyte tissue volume in bone marrow is increased with aging and in patients with osteoporosis. Biogerontology.

[25] Verma S., Rajaratnam J., Denton J., Hoyland J., Byers R. (2002). Adipocytic proportion of bone marrow is inversely related to bone formation in osteoporosis. J. Clin. Pathol..

[26] Moerman E.J., Teng K., Lipschitz D.A., Lecka-Czernik B. (2004). Aging activates adipogenic and suppresses osteogenic programs in mesenchymal marrow stroma/stem cells: The role of PPAR-γ2 transcription factor and TGF-β/BMP signaling pathways. Aging Cell.

[27] Baker N., Boyette L.B., Tuan R.S. (2015). Characterization of bone marrow-derived mesenchymal stem cells in aging. Bone.

[28] Beane O.S., Fonseca V.C., Cooper L.L., Koren G., Darling E.M. (2014). Impact of aging on the regenerative properties of bone marrow-, muscle-, and adipose-derived mesenchymal stem/stromal cells. PLoS ONE.

[29] Mattiucci D., Maurizi G., Leoni P., Poloni A. (2018). Aging-and Senescence-associated Changes of Mesenchymal Stromal Cells in Myelodysplastic Syndromes. Cell Transplant..

[30] Turinetto V., Vitale E., Giachino C. (2016). Senescence in human mesenchymal stem cells: Functional changes and implications in stem cell-based therapy. Int. J. Mol. Sci..

[31] Fathi E., Charoudeh H.N., Sanaat Z., Farahzadi R. (2019). Telomere shortening as a hallmark of stem cell senescence. Stem Cell Investig..

[32] Galderisi U., Helmbold H., Squillaro T., Alessio N., Komm N., Khadang B., Cipollaro M., Bohn W., Giordano A. (2009). In vitro senescence of rat mesenchymal stem cells is accompanied by downregulation of stemness-related and DNA damage repair genes. Stem Cells Dev..

[33] Vono R., Jover Garcia E., Spinetti G., Madeddu P. (2018). Oxidative stress in mesenchymal stem cell senescence: Regulation by coding and noncoding RNAs. Antioxid. Redox Signal..

[34] Roninson I.B. (2002). Oncogenic functions of tumour suppressor p21Waf1/Cip1/Sdi1: Association with cell senescence and tumour-promoting activities of stromal fibroblasts. Cancer Lett..

[35] Kang C., Xu Q., Martin T.D., Li M.Z., Demaria M., Aron L., Lu T., Yankner B.A., Campisi J., Elledge S.J. (2015). The DNA damage response induces inflammation and senescence by inhibiting autophagy of GATA4. Science.

[36] Acosta J.C., O’Loghlen A., Banito A., Guijarro M.V., Augert A., Raguz S., Fumagalli M., Da Costa M., Brown C., Popov N. (2008). Chemokine signaling via the CXCR2 receptor reinforces senescence. Cell.

[37] Kuilman T., Michaloglou C., Vredeveld L.C., Douma S., van Doorn R., Desmet C.J., Aarden L.A., Mooi W.J., Peeper D.S. (2008). Oncogene-induced senescence relayed by an interleukin-dependent inflammatory network. Cell.

[38] Kim M., Kim C., Choi Y.S., Kim M., Park C., Suh Y. (2012). Age-related alterations in mesenchymal stem cells related to shift in differentiation from osteogenic to adipogenic potential: Implication to age-associated bone diseases and defects. Mech. Ageing Dev..

[39] Sepúlveda J.C., Tomé M., Fernández M.E., Delgado M., Campisi J., Bernad A., González M.A. (2014). Cell senescence abrogates the therapeutic potential of human mesenchymal stem cells in the lethal endotoxemia model. Stem Cells.

[40] Li Y., Xu X., Wang L., Liu G., Li Y., Wu X., Jing Y., Li H., Wang G. (2015). Senescent mesenchymal stem cells promote colorectal cancer cells growth via galectin-3 expression. Cell Biosci..

[41] Skolekova S., Matuskova M., Bohac M., Toro L., Durinikova E., Tyciakova S., Demkova L., Gursky J., Kucerova L. (2016). Cisplatin-induced mesenchymal stromal cells-mediated mechanism contributing to decreased antitumor effect in breast cancer cells. Cell Commun. Signal..

[42] Farr J.N., Fraser D.G., Wang H., Jaehn K., Ogrodnik M.B., Weivoda M.M., Drake M.T., Tchkonia T., LeBrasseur N.K., Kirkland J.L. (2016). Identification of senescent cells in the bone microenvironment. J. Bone Miner. Res..

[43] Geyh S., Öz S., Cadeddu R., Fröbel J., Brückner B., Kündgen A., Fenk R., Bruns I., Zilkens C., Hermsen D. (2013). Insufficient stromal support in MDS results from molecular and functional deficits of mesenchymal stromal cells. Leukemia.

[44] Wang D., Jang D.-J. (2009). Protein Kinase CK2 Regulates Cytoskeletal Reorganization during Ionizing Radiation–Induced Senescence of Human Mesenchymal Stem Cells. Cancer Res..

[45] Wang E. (1995). Senescent human fibroblasts resist programmed cell death, and failure to suppress bcl2 is involved. Cancer Res..

[46] Almalki S.G., Agrawal D.K. (2016). Key transcription factors in the differentiation of mesenchymal stem cells. Differentiation.

[47] Varela N., Aranguiz A., Lizama C., Sepulveda H., Antonelli M., Thaler R., Moreno R.D., Montecino M., Stein G.S., Van Wijnen A.J. (2016). Mitotic inheritance of mRNA facilitates translational activation of the osteogenic-Lineage commitment factor runx2 in progeny of osteoblastic cells. J. Cell. Physiol..

[48] Ducy P., Zhang R., Geoffroy V., Ridall A.L., Karsenty G. (1997). Osf2/Cbfa1: A transcriptional activator of osteoblast differentiation. Cell.

[49] Yang D.C., Yang M.H., Tsai C.-C., Huang T.F., Chen Y.H., Hung S.C. (2011). Hypoxia inhibits osteogenesis in human mesenchymal stem cells through direct regulation of RUNX2 by TWIST. PLoS ONE.

[50] Jiang Y., Mishima H., Sakai S., Liu Y.K., Ohyabu Y., Uemura T. (2008). Gene expression analysis of major lineage-defining factors in human bone marrow cells: Effect of aging, gender, and age-related disorders. J. Orthop. Res..

[51] Nakashima K., Zhou X., Kunkel G., Zhang Z., Deng J.M., Behringer R.R., de Crombrugghe B. (2002). The novel zinc finger-containing transcription factor osterix is required for osteoblast differentiation and bone formation. Cell.

[52] Querques F., D’Agostino A., Cozzolino C., Cozzuto L., Lombardo B., Leggiero E., Ruosi C., Pastore L. (2019). Identification of a Novel Transcription Factor Required for Osteogenic Differentiation of Mesenchymal Stem Cells. Stem Cells Dev..

[53] Davis K.E., Moldes M., Farmer S.R. (2004). The forkhead transcription factor FoxC2 inhibits white adipocyte differentiation. J. Biol. Chem..

[54] Okitsu Y., Takahashi S., Minegishi N., Kameoka J., Kaku M., Yamamoto M., Sasaki T., Harigae H. (2007). Regulation of adipocyte differentiation of bone marrow stromal cells by transcription factor GATA-2. Biochem. Biophys. Res. Commun..

[55] Seifert A., Werheid D.F., Knapp S.M., Tobiasch E. (2015). Role of Hox genes in stem cell differentiation. World J. Stem Cells.

[56] La Cour Poulsen L., Siersbæk M., Mandrup S. (2012). PPARs: Fatty acid sensors controlling metabolism. Semin. Cell Dev. Biol..

[57] Bionaz M., Monaco E., Wheeler M.B. (2015). Transcription adaptation during in vitro adipogenesis and osteogenesis of porcine mesenchymal stem cells: Dynamics of pathways, biological processes, up-stream regulators, and gene networks. PLoS ONE.

[58] Zhu Y., Qi C., Korenberg J.R., Chen X.-N., Noya D., Rao M.S., Reddy J.K. (1995). Structural organization of mouse peroxisome proliferator-activated receptor gamma (mPPAR gamma) gene: Alternative promoter use and different splicing yield two mPPAR gamma isoforms. Proc. Natl. Acad. Sci. USA.

[59] Yu W.H., Li F.G., Chen X.Y., Li J.T., Wu Y.H., Huang L.H., Wang Z., Li P., Wang T., Lahn B.T. (2012). PPARγ suppression inhibits adipogenesis but does not promote osteogenesis of human mesenchymal stem cells. Int. J. Biochem. Cell Biol..

[60] Hesslein D.G., Fretz J.A., Xi Y., Nelson T., Zhou S., Lorenzo J.A., Schatz D.G., Horowitz M.C. (2009). Ebf1-dependent control of the osteoblast and adipocyte lineages. Bone.

[61] Poyton R.O., Ball K.A., Castello P.R. (2009). Mitochondrial generation of free radicals and hypoxic signaling. Trends Endocrinol. Metab..

[62] Milani P., Ambrosi G., Gammoh O., Blandini F., Cereda C. (2013). SOD1 and DJ-1 converge at Nrf2 pathway: A clue for antioxidant therapeutic potential in neurodegeneration. Oxidative Med. Cell. Longev..

[63] Li H., Liu P., Xu S., Li Y., Dekker J.D., Li B., Fan Y., Zhang Z., Hong Y., Yang G. (2017). FOXP1 controls mesenchymal stem cell commitment and senescence during skeletal aging. J. Clin. Investig..

[64] Chen G., Deng C., Li Y.-P. (2012). TGF-β and BMP signaling in osteoblast differentiation and bone formation. Int. J. Biol. Sci..

[65] Freire M.O., You H.-K., Kook J.-K., Choi J.-H., Zadeh H.H. (2011). Antibody-mediated osseous regeneration: A novel strategy for bioengineering bone by immobilized anti–bone morphogenetic protein-2 antibodies. Tissue Eng. Part. A.

[66] Kang Q., Song W.-X., Luo Q., Tang N., Luo J., Luo X., Chen J., Bi Y., He B.C., Park J.K. (2008). A comprehensive analysis of the dual roles of BMPs in regulating adipogenic and osteogenic differentiation of mesenchymal progenitor cells. Stem Cells Dev..

[67] Tang Q.-Q., Otto T.C., Lane M.D. (2004). Commitment of C3H10T1/2 pluripotent stem cells to the adipocyte lineage. Proc. Natl. Acad. Sci. USA.

[68] Zur Nieden N.I., Kempka G., Rancourt D.E., Ahr H.-J. (2005). Induction of chondro-, osteo-and adipogenesis in embryonic stem cells by bone morphogenetic protein-2: Effect of cofactors on differentiating lineages. BMC Dev. Biol..

[69] Deng Z.L., Sharff K.A., Tang N., Song W.X., Luo J., Luo X., Chen J., Bennett E., Reid R., Manning D. (2008). Regulation of osteogenic differentiation during skeletal development. Front. Biosci..

[70] Miyazono K., Maeda S., Imamura T. (2005). BMP receptor signaling: Transcriptional targets, regulation of signals, and signaling cross-talk. Cytokine Growth Factor Rev..

[71] Li X., Cao X. (2006). BMP signaling and skeletogenesis. Ann. N. Y. Acad. Sci..

[72] Niehrs C. (2012). The complex world of WNT receptor signalling. Nat. Rev. Mol. Cell Biol..

[73] White B.D., Chien A.J., Dawson D.W. (2012). Dysregulation of Wnt/β-catenin signaling in gastrointestinal cancers. Gastroenterology.

[74] Clevers H., Loh K.M., Nusse R. (2014). An integral program for tissue renewal and regeneration: Wnt signaling and stem cell control. Science.

[75] Muruganandan S., Roman A., Sinal C. (2009). Adipocyte differentiation of bone marrow-derived mesenchymal stem cells: Cross talk with the osteoblastogenic program. Cell. Mol. Life Sci..

[76] Byun M., Hwang J., Kim A., Kim K., Hwang E., Yaffe M., Hong J.-H. (2014). Canonical Wnt signalling activates TAZ through PP1A during osteogenic differentiation. Cell Death Differ..

[77] Bennett C.N., Ouyang H., Ma Y.L., Zeng Q., Gerin I., Sousa K.M., Lane T.F., Krishnan V., Hankenson K.D., MacDougald O.A. (2007). Wnt10b increases postnatal bone formation by enhancing osteoblast differentiation. J. Bone Miner. Res..

[78] Stevens J.R., Miranda-Carboni G.A., Singer M.A., Brugger S.M., Lyons K.M., Lane T.F. (2010). Wnt10b deficiency results in age-dependent loss of bone mass and progressive reduction of mesenchymal progenitor cells. J. Bone Miner. Res..

[79] Arango N.A., Szotek P.P., Manganaro T.F., Oliva E., Donahoe P.K., Teixeira J. (2005). Conditional deletion of β-catenin in the mesenchyme of the developing mouse uterus results in a switch to adipogenesis in the myometrium. Dev. Biol..

[80] Lin G.L., Hankenson K.D. (2011). Integration of BMP, Wnt, and notch signaling pathways in osteoblast differentiation. J. Cell. Biochem..

[81] Shimizu T., Tanaka T., Iso T., Matsui H., Ooyama Y., Kawai-Kowase K., Arai M., Kurabayashi M. (2011). Notch signaling pathway enhances bone morphogenetic protein 2 (BMP2) responsiveness of Msx2 gene to induce osteogenic differentiation and mineralization of vascular smooth muscle cells. J. Biol. Chem..

[82] Spinella-Jaegle S., Rawadi G., Kawai S., Gallea S., Faucheu C., Mollat P., Courtois B., Bergaud B., Ramez V., Blanchet A.M. (2001). Sonic hedgehog increases the commitment of pluripotent mesenchymal cells into the osteoblastic lineage and abolishes adipocytic differentiation. J. Cell Sci..

[83] James A.W., Pan A., Chiang M., Zara J.N., Zhang X., Ting K., Soo C. (2011). A new function of Nell-1 protein in repressing adipogenic differentiation. Biochem. Biophys. Res. Commun..

[84] Zhang X., Zara J., Siu R., Ting K., Soo C. (2010). The role of NELL-1, a growth factor associated with craniosynostosis, in promoting bone regeneration. J. Dent. Res..

[85] Neubauer M., Fischbach C., Bauer-Kreisel P., Lieb E., Hacker M., Tessmar J., Schulz M.B., Goepferich A., Blunk T. (2004). Basic fibroblast growth factor enhances PPARγ ligand-induced adipogenesis of mesenchymal stem cells. FEBS Lett..

[86] Woei Ng K., Speicher T., Dombrowski C., Helledie T., Haupt L.M., Nurcombe V., Cool S.M. (2007). Osteogenic differentiation of murine embryonic stem cells is mediated by fibroblast growth factor receptors. Stem Cells Dev..

[87] Giustina A., Mazziotti G., Canalis E. (2008). Growth hormone, insulin-like growth factors, and the skeleton. Endocr. Rev..

[88] Hayflick L., Moorhead P.S. (1961). The serial cultivation of human diploid cell strains. Exp. Cell Res..

[89] Di Fagagna F.d.A. (2008). Living on a break: Cellular senescence as a DNA-damage response. Nat. Rev. Cancer.

[90] Shay J.W., Wright W.E. (2004). Senescence and immortalization: Role of telomeres and telomerase. Carcinogenesis.

[91] Moussavi-Harami F., Duwayri Y., Martin J.A., Moussavi-Harami F., Buckwalter J.A. (2004). Oxygen effects on senescence in chondrocytes and mesenchymal stem cells: Consequences for tissue engineering. Iowa Orthop. J..

[92] Wu J., Niu J., Li X., Wang X., Guo Z., Zhang F. (2014). TGF-β1 induces senescence of bone marrow mesenchymal stem cells via increase of mitochondrial ROS production. BMC Dev. Biol..

[93] Gu Z., Tan W., Feng G., Meng Y., Shen B., Liu H., Cheng C. (2014). Wnt/β-catenin signaling mediates the senescence of bone marrow-mesenchymal stem cells from systemic lupus erythematosus patients through the p53/p21 pathway. Mol. Cell. Biochem..

[94] Wang Y., Chen S., Yan Z., Pei M. (2019). A prospect of cell immortalization combined with matrix microenvironmental optimization strategy for tissue engineering and regeneration. Cell Biosci..

[95] Komori T. (2006). Regulation of osteoblast differentiation by transcription factors. J. Cell. Biochem..

[96] Westendorf J.J. (2006). Transcriptional co-repressors of Runx2. J. Cell. Biochem..

[97] Stein G.S., Lian J.B., van Wijnen A.J., Stein J.L. (1997). The osteocalcin gene: A model for multiple parameters of skeletal-specific transcriptional control. Mol. Biol. Rep..

[98] Shen J., Hovhannisyan H., Lian J.B., Montecino M.A., Stein G.S., Stein J.L., Van Wijnen A.J. (2003). Transcriptional induction of the osteocalcin gene during osteoblast differentiation involves acetylation of histones h3 and h4. Mol. Endocrinol..

[99] Villar-Garea A., Esteller M. (2004). Histone deacetylase inhibitors: Understanding a new wave of anticancer agents. Int. J. Cancer.

[100] Villagra A., Gutiérrez J., Paredes R., Sierra J., Puchi M., Imschenetzky M., Van Wijnen A., Lian J., Stein G., Stein J. (2002). Reduced CpG methylation is associated with transcriptional activation of the bone-specific rat osteocalcin gene in osteoblasts. J. Cell. Biochem..

[101] Arnsdorf E.J., Tummala P., Castillo A.B., Zhang F., Jacobs C.R. (2010). The epigenetic mechanism of mechanically induced osteogenic differentiation. J. Biomech..

[102] Ling M., Huang P., Islam S., Heruth D.P., Li X., Zhang L.Q., Li D.-Y., Hu Z., Ye S.Q. (2017). Epigenetic regulation of Runx2 transcription and osteoblast differentiation by nicotinamide phosphoribosyltransferase. Cell Biosci..

[103] Tominaga H., Maeda S., Hayashi M., Takeda S., Akira S., Komiya S., Nakamura T., Akiyama H., Imamura T. (2008). CCAAT/enhancer-binding protein β promotes osteoblast differentiation by enhancing Runx2 activity with ATF4. Mol. Biol. Cell.

[104] Yin B., Yu F., Wang C., Li B., Liu M., Ye L. (2019). Epigenetic Control of Mesenchymal Stem Cell Fate Decision via Histone Methyltransferase Ash1l. Stem Cells.

[105] Baglìo S.R., Devescovi V., Granchi D., Baldini N. (2013). MicroRNA expression profiling of human bone marrow mesenchymal stem cells during osteogenic differentiation reveals Osterix regulation by miR-31. Gene.

[106] Eskildsen T., Taipaleenmäki H., Stenvang J., Abdallah B.M., Ditzel N., Nossent A.Y., Bak M., Kauppinen S., Kassem M. (2011). MicroRNA-138 regulates osteogenic differentiation of human stromal (mesenchymal) stem cells in vivo. Proc. Natl. Acad. Sci. USA.

[107] Huang J., Zhao L., Xing L., Chen D. (2010). MicroRNA-204 regulates Runx2 protein expression and mesenchymal progenitor cell differentiation. Stem Cells.

[108] Zhang J., Fu W.M., He M.L., Wang H., Wang W.M., Yu S.C., Bian X.W., Zhou J., Lin M.C., Lu G. (2011). MiR-637 maintains the balance between adipocytes and osteoblasts by directly targeting Osterix. Mol. Biol. Cell.

[109] Yan Z., Guo Y., Wang Y., Li Y., Wang J. (2018). MicroRNA profiles of BMSCs induced into osteoblasts with osteoinductive medium. Exp. Ther. Med..

[110] Morrison R.F., Farmer S.R. (1999). Insights into the transcriptional control of adipocyte differentiation. J. Cell. Biochem..

[111] Noer A., Sørensen A.L., Boquest A.C., Collas P. (2006). Stable CpG hypomethylation of adipogenic promoters in freshly isolated, cultured, and differentiated mesenchymal stem cells from adipose tissue. Mol. Biol. Cell.

[112] Bowers R.R., Kim J.W., Otto T.C., Lane M.D. (2006). Stable stem cell commitment to the adipocyte lineage by inhibition of DNA methylation: Role of the BMP-4 gene. Proc. Natl. Acad. Sci. USA.

[113] Musri M.M., Corominola H., Casamitjana R., Gomis R., Párrizas M. (2006). Histone H3 lysine 4 dimethylation signals the transcriptional competence of the adiponectin promoter in preadipocytes. J. Biol. Chem..

[114] Fajas L., Egler V., Reiter R., Hansen J., Kristiansen K., Debril M.-B., Miard S., Auwerx J. (2002). The retinoblastoma-histone deacetylase 3 complex inhibits PPARγ and adipocyte differentiation. Dev. Cell.

[115] Qadir A.S., Woo K.M., Ryoo H.-M., Baek J.-H. (2013). Insulin suppresses distal-less homeobox 5 expression through the up-regulation of microRNA-124 in 3T3-L1 cells. Exp. Cell Res..

[116] Hamam D., Ali D., Vishnubalaji R., Hamam R., Al-Nbaheen M., Chen L., Kassem M., Aldahmash A., Alajez N.M. (2014). microRNA-320/RUNX2 axis regulates adipocytic differentiation of human mesenchymal (skeletal) stem cells. Cell Death Amp. Dis..

[117] Zaragosi L.-E., Wdziekonski B., Brigand K.L., Villageois P., Mari B., Waldmann R., Dani C., Barbry P. (2011). Small RNA sequencing reveals miR-642a-3p as a novel adipocyte-specific microRNA and miR-30 as a key regulator of human adipogenesis. Genome Biol..

[118] Li C.J., Cheng P., Liang M.K., Chen Y.S., Lu Q., Wang J.Y., Xia Z.Y., Zhou H.D., Cao X., Xie H. (2015). MicroRNA-188 regulates age-related switch between osteoblast and adipocyte differentiation. J. Clin. Investig..

[119] Nilsson O., Mitchum R.D., Schrier L., Ferns S.P., Barnes K.M., Troendle J.F., Baron J. (2005). Growth plate senescence is associated with loss of DNA methylation. J. Endocrinol..

[120] So A.Y., Jung J.W., Lee S., Kim H.S., Kang K.S. (2011). DNA methyltransferase controls stem cell aging by regulating BMI1 and EZH2 through microRNAs. PLoS ONE.

[121] Jung J.W., Lee S., Seo M.S., Park S.B., Kurtz A., Kang S.K., Kang K.S. (2010). Histone deacetylase controls adult stem cell aging by balancing the expression of polycomb genes and jumonji domain containing 3. Cell Mol. Life Sci..

[122] Fernandez A.F., Bayon G.F., Urdinguio R.G., Torano E.G., Garcia M.G., Carella A., Petrus-Reurer S., Ferrero C., Martinez-Camblor P., Cubillo I. (2015). H3K4me1 marks DNA regions hypomethylated during aging in human stem and differentiated cells. Genome Res..

[123] Yoo J.K., Kim J., Choi S.J., Noh H.M., Kwon Y.D., Yoo H., Yi H.S., Chung H.M., Kim J.K. (2012). Discovery and characterization of novel microRNAs during endothelial differentiation of human embryonic stem cells. Stem Cells Dev..

[124] Benhamed M., Herbig U., Ye T., Dejean A., Bischof O. (2012). Senescence is an endogenous trigger for microRNA-directed transcriptional gene silencing in human cells. Nat. Cell Biol..

[125] Lal A., Kim H.H., Abdelmohsen K., Kuwano Y., Pullmann R., Srikantan S., Subrahmanyam R., Martindale J.L., Yang X., Ahmed F. (2008). p16(INK4a) translation suppressed by miR-24. PLoS ONE.

[126] Wagner W., Horn P., Castoldi M., Diehlmann A., Bork S., Saffrich R., Benes V., Blake J., Pfister S., Eckstein V. (2008). Replicative senescence of mesenchymal stem cells: A continuous and organized process. PLoS ONE.

[127] Yamakuchi M., Lowenstein C.J. (2009). MiR-34, SIRT1 and p53: The feedback loop. Cell Cycle.

[128] Li X., Song Y., Liu D., Zhao J., Xu J., Ren J., Hu Y., Wang Z., Hou Y., Zhao G. (2017). MiR-495 Promotes Senescence of Mesenchymal Stem Cells by Targeting Bmi-1. Cell Physiol. Biochem..

[129] Okada M., Kim H.W., Matsu-ura K., Wang Y.G., Xu M., Ashraf M. (2016). Abrogation of Age-Induced MicroRNA-195 Rejuvenates the Senescent Mesenchymal Stem Cells by Reactivating Telomerase. Stem Cells.

[130] Coelho M., Fernandes M. (2000). Human bone cell cultures in biocompatibility testing. Part II: Effect of ascorbic acid, β-glycerophosphate and dexamethasone on osteoblastic differentiation. Biomaterials.

[131] Pittenger M.F., Mackay A.M., Beck S.C., Jaiswal R.K., Douglas R., Mosca J.D., Moorman M.A., Simonetti D.W., Craig S., Marshak D.R. (1999). Multilineage potential of adult human mesenchymal stem cells. Science.

[132] Brindle P.K., Montminy M.R. (1992). The CREB family of transcription activators. Curr. Opin. Genet. Dev..

[133] Dimitriadis G., Mitrou P., Lambadiari V., Maratou E., Raptis S.A. (2011). Insulin effects in muscle and adipose tissue. Diabetes Res. Clin. Pract..

[134] Chang T.-C., Hsu M.-F., Wu K.K. (2015). High glucose induces bone marrow-derived mesenchymal stem cell senescence by upregulating autophagy. PLoS ONE.

[135] Hochane M., Trichet V., Pecqueur C., Avril P., Oliver L., Denis J., Brion R., Amiaud J., Pineau A., Naveilhan P. (2017). Low-Dose Pesticide Mixture Induces Senescence in Normal Mesenchymal Stem Cells (MSC) and Promotes Tumorigenic Phenotype in Premalignant MSC. Stem Cells.

[136] Islam M.S., Stemig M.E., Takahashi Y., Hui S.K. (2015). Radiation response of mesenchymal stem cells derived from bone marrow and human pluripotent stem cells. J. Radiat Res..

[137] Alessio N., Del Gaudio S., Capasso S., Di Bernardo G., Cappabianca S., Cipollaro M., Peluso G., Galderisi U. (2014). Low dose radiation induced senescence of human mesenchymal stromal cells and impaired the autophagy process. Oncotarget.

[138] Menuki K., Mori T., Sakai A., Sakuma M., Okimoto N., Shimizu Y., Kunugita N., Nakamura T. (2008). Climbing exercise enhances osteoblast differentiation and inhibits adipogenic differentiation with high expression of PTH/PTHrP receptor in bone marrow cells. Bone.

[139] Demiray L., Ozcivici E. (2015). Bone Marrow Stem Cells Adapt to Low-Magnitude Vibrations by Altering Theircytoskeleton During Quiescence and. Turk. J. Biol..

[140] Zayzafoon M., Gathings W.E., McDonald J.M. (2004). Modeled microgravity inhibits osteogenic differentiation of human mesenchymal stem cells and increases adipogenesis. Endocrinology.

[141] Tormos K.V., Anso E., Hamanaka R.B., Eisenbart J., Joseph J., Kalyanaraman B., Chandel N.S. (2011). Mitochondrial complex III ROS regulate adipocyte differentiation. Cell Metab..

[142] Da Silva S.V., Renovato-Martins M., Ribeiro-Pereira C., Citelli M., Barja-Fidalgo C. (2016). Obesity modifies bone marrow microenvironment and directs bone marrow mesenchymal cells to adipogenesis. Obesity.

[143] Campisi J. (2001). From cells to organisms: Can we learn about aging from cells in culture?. Exp. Gerontol..

[144] Phetfong J., Sanvoranart T., Nartprayut K., Nimsanor N., Seenprachawong K., Prachayasittikul V., Supokawej A. (2016). Osteoporosis: The current status of mesenchymal stem cell-based therapy. Cell. Mol. Biol. Lett..

[145] Ye X., Zhang P., Xue S., Xu Y., Tan J., Liu G. (2014). Adipose-derived stem cells alleviate osteoporosis by enchancing osteogenesis and inhibiting adipogenesis in a rabbit model. Cytotherapy.

[146] Ichioka N., Inaba M., Kushida T., Esumi T., Takahara K., Inaba K., Ogawa R., Iida H., Ikehara S. (2002). Prevention of senile osteoporosis in SAMP6 mice by intrabone marrow injection of allogeneic bone marrow cells. Stem Cells.

[147] Takada K., Inaba M., Ichioka N., Ueda Y., Taira M., Baba S., Mizokami T., Wang X., Hisha H., Iida H. (2006). Treatment of senile osteoporosis in SAMP6 mice by intra-bone marrow injection of allogeneic bone marrow cells. Stem Cells.

[148] Ocarino Nde M., Boeloni J.N., Jorgetti V., Gomes D.A., Goes A.M., Serakides R. (2010). Intra-bone marrow injection of mesenchymal stem cells improves the femur bone mass of osteoporotic female rats. Connect. Tissue Res..

[149] Kiernan J., Hu S., Grynpas M.D., Davies J.E., Stanford W.L. (2016). Systemic Mesenchymal Stromal Cell Transplantation Prevents Functional Bone Loss in a Mouse Model of Age-Related Osteoporosis. Stem Cells Transl. Med..

[150] An Q., Wu D., Ma Y., Zhou B., Liu Q. (2015). Suppression of Evi1 promotes the osteogenic differentiation and inhibits the adipogenic differentiation of bone marrow-derived mesenchymal stem cells in vitro. Int. J. Mol. Med..

[151] Jing H., Liao L., An Y., Su X., Liu S., Shuai Y., Zhang X., Jin Y. (2016). Suppression of EZH2 prevents the shift of osteoporotic MSC fate to adipocyte and enhances bone formation during osteoporosis. Mol. Ther..

[152] Zhou X., Liu Z., Huang B., Yan H., Yang C., Li Q., Jin D. (2019). Orcinol glucoside facilitates the shift of MSC fate to osteoblast and prevents adipogenesis via Wnt/β-catenin signaling pathway. Drug Des. Dev. Ther..

[153] Wei J., Li H., Wang S., Li T., Fan J., Liang X., Li J., Han Q., Zhu L., Fan L. (2014). let-7 enhances osteogenesis and bone formation while repressing adipogenesis of human stromal/mesenchymal stem cells by regulating HMGA2. Stem Cells Dev..

[154] Zhao Z., Li X., Zou D., Lian Y., Tian S., Dou Z. (2019). Expression of microRNA-21 in osteoporotic patients and its involvement in the regulation of osteogenic differentiation. Exp. Ther. Med..

[155] Li C.-J., Xiao Y., Yang M., Su T., Sun X., Guo Q., Huang Y., Luo X.-H. (2018). Long noncoding RNA Bmncr regulates mesenchymal stem cell fate during skeletal aging. J. Clin. Investig..

[156] Chen X., Yang L., Ge D., Wang W., Yin Z., Yan J., Cao X., Jiang C., Zheng S., Liang B. (2019). Long non‑coding RNA XIST promotes osteoporosis through inhibiting bone marrow mesenchymal stem cell differentiation. Exp. Ther. Med..

[157] Zhu X., Yu J., Du J., Zhong G., Qiao L., Lin J. (2019). LncRNA HOXA-AS2 positively regulates osteogenesis of mesenchymal stem cells through inactivating NF-κB signalling. J. Cell. Mol. Med..

[158] Kim H.N., Chang J., Shao L., Han L., Iyer S., Manolagas S.C., O’Brien C.A., Jilka R.L., Zhou D., Almeida M. (2017). DNA damage and senescence in osteoprogenitors expressing Osx1 may cause their decrease with age. Aging Cell.

[159] Gao B., Lin X., Jing H., Fan J., Ji C., Jie Q., Zheng C., Wang D., Xu X., Hu Y. (2018). Local delivery of tetramethylpyrazine eliminates the senescent phenotype of bone marrow mesenchymal stromal cells and creates an anti-inflammatory and angiogenic environment in aging mice. Aging Cell.

[160] Sun J., Ming L., Shang F., Shen L., Chen J., Jin Y. (2015). Apocynin suppression of NADPH oxidase reverses the aging process in mesenchymal stem cells to promote osteogenesis and increase bone mass. Sci. Rep..

[161] Zhou T., Yan Y., Zhao C., Xu Y., Wang Q., Xu N. (2019). Resveratrol improves osteogenic differentiation of senescent bone mesenchymal stem cells through inhibiting endogenous reactive oxygen species production via AMPK activation. Redox Rep..

